# Snapshot of iron response in *Shewanella oneidensis *by gene network reconstruction

**DOI:** 10.1186/1471-2164-10-131

**Published:** 2009-03-25

**Authors:** Yunfeng Yang, Daniel P Harris, Feng Luo, Wenlu Xiong, Marcin Joachimiak, Liyou Wu, Paramvir Dehal, Janet Jacobsen, Zamin Yang, Anthony V Palumbo, Adam P Arkin, Jizhong Zhou

**Affiliations:** 1 Biosciences Division, Oak Ridge National Laboratory, Oak Ridge, TN, 37831, USA; 2 School of Computing, Clemson University, Clemson, SC, 29634, USA; 3 Physical Biosciences Division, Lawrence Berkeley National Laboratory, Berkeley, CA, 94720, USA; 4 Institute for Environmental Genomics, and Department of Botany and Microbiology, University of Oklahoma, Norman, OK, 73019, USA; 5 Computational Research Division, Lawrence Berkeley National Laboratory, Berkeley, CA, 94720, USA; 6 Department of Bioengineering, University of California, Berkeley, CA, 94720, USA

## Abstract

**Background:**

Iron homeostasis of *Shewanella oneidensis*, a γ-proteobacterium possessing high iron content, is regulated by a global transcription factor Fur. However, knowledge is incomplete about other biological pathways that respond to changes in iron concentration, as well as details of the responses. In this work, we integrate physiological, transcriptomics and genetic approaches to delineate the iron response of *S. oneidensis*.

**Results:**

We show that the iron response in *S. oneidensis *is a rapid process. Temporal gene expression profiles were examined for iron depletion and repletion, and a gene co-expression network was reconstructed. Modules of iron acquisition systems, anaerobic energy metabolism and protein degradation were the most noteworthy in the gene network. Bioinformatics analyses suggested that genes in each of the modules might be regulated by DNA-binding proteins Fur, CRP and RpoH, respectively. Closer inspection of these modules revealed a transcriptional regulator (SO2426) involved in iron acquisition and ten transcriptional factors involved in anaerobic energy metabolism. Selected genes in the network were analyzed by genetic studies. Disruption of genes encoding a putative alcaligin biosynthesis protein (SO3032) and a gene previously implicated in protein degradation (SO2017) led to severe growth deficiency under iron depletion conditions. Disruption of a novel transcriptional factor (SO1415) caused deficiency in both anaerobic iron reduction and growth with thiosulfate or TMAO as an electronic acceptor, suggesting that SO1415 is required for specific branches of anaerobic energy metabolism pathways.

**Conclusion:**

Using a reconstructed gene network, we identified major biological pathways that were differentially expressed during iron depletion and repletion. Genetic studies not only demonstrated the importance of iron acquisition and protein degradation for iron depletion, but also characterized a novel transcriptional factor (SO1415) with a role in anaerobic energy metabolism.

## Background

Iron is an important nutrient for bacteria, serving as a co-factor for proteins involved in respiration, the tricarboxylic acid (TCA) cycle, enzyme catalysis, gene regulation, photosynthesis, N_2 _fixation, methanogenesis, H_2 _production and consumption, oxygen transport, and DNA biosynthesis [1-3]. Iron exists in two redox states under physiological conditions: the insoluble Fe(III) ferric form and the relatively soluble Fe(II) ferrous form, which renders it a useful enzyme prosthetic group [4]. However, iron can induce oxidative stress by catalyzing Fenton reactions, and the prevalent Fe(III) form has low bioavailability [4,5]. Intracellular levels of iron must, therefore, be carefully controlled to meet the metabolic needs of the cell while limiting cellular damage due to iron overload [6-8].

Microbial iron response has been well-studied in *Escherichia coli*, which adapts to low iron conditions by inducing iron-binding transporters embedded in the outer and inner membranes to import iron [1,9]. Iron-chelating siderophores also are secreted to solubilize iron prior to transportation across the membranes [4]. Concurrently, production of non-essential proteins that use iron is inhibited, which increases the pool of free iron in the cell [10]. Iron homeostasis in a diverse group of prokaryotes is maintained by the global transcriptional factor Fur (Ferric Uptake Regulator) and small regulatory RNA RyhB. Fur is an iron-responsive protein dimer with an amino-terminal helix-turn-helix DNA binding domain and a metal-binding domain [1,11]. Each Fur monomer complexes with Fe(II) and binds the major groove of iron responsive gene promoters at a conserved 19-bp inverted repeat sequence (GATAATGATAATCATTATC) called the "Fur box". The Fur box, located near the cognate promoter [11,12], effectively prevents recruitment of RNA polymerase holoenzyme to the promoter and thus represses transcription [13,14]. Fur directly represses the expression of RyhB small RNA, which in turn represses a large group of mRNAs encoding non-essential, iron-using proteins [10,15]. This regulatory cascade of Fur and RyhB is believed to ensure that iron is directed towards essential iron-using proteins during iron limitation [16].

Studying iron homeostasis in *S. oneidensis *is of interest for a number of reasons. Known terminal electron acceptors of *S. oneidensis *include a variety of inorganic and organic compounds such as fumarate, nitrate, thiosulfate, trimethylamine N-oxide (TMAO), Fe(III), Mn(IV), Cr(VI), and U(VI) [17]. The ability of *S. oneidensis *to respire Fe(III) is unique among the known γ-proteobacteria. Iron not only acts as a cofactor in *S. oneidensis*, but also as an important terminal electron acceptor. *S. oneidensis *is also striking for its high cellular demand for iron due to a high cellular content of heme [18], which is a protein cofactor requiring iron. The characteristic pink or red color of the cells indicates the high content of heme in *S. oneidensis*. In addition, *S. oneidensis *has been shown to be a fish pathogen as well as an opportunistic human pathogen [19,20]. Iron-regulated metabolisms are essential for the virulence of *Vibrio cholerae *[21], *Bacillus cereus *[22], *Neisseria meningitidis *[23] and *Shigella *species [24]. It is possible that iron may play a similar role in *S. oneidensis *as it does in other pathogenic γ-proteobacteria (e.g. *Vibrio *and *Shigella*).

An ortholog of Fur has been identified in *S. oneidensis *[2]. Its major role is to act as a repressor that mediates transcription of genes involved in siderophore biosynthesis and iron acquisition systems [2,3,25] and hence plays a role in response to iron depletion. It remains unclear, however, which other biological pathways respond to the change of iron concentration, as well as the nature of those responses. To identify iron-responsive processes, we carried out an integrative study with physiological, transcriptomic and genetic approaches.

## Results

### Physiological response of MR-1 to changes in iron concentration

**Figure 1:**
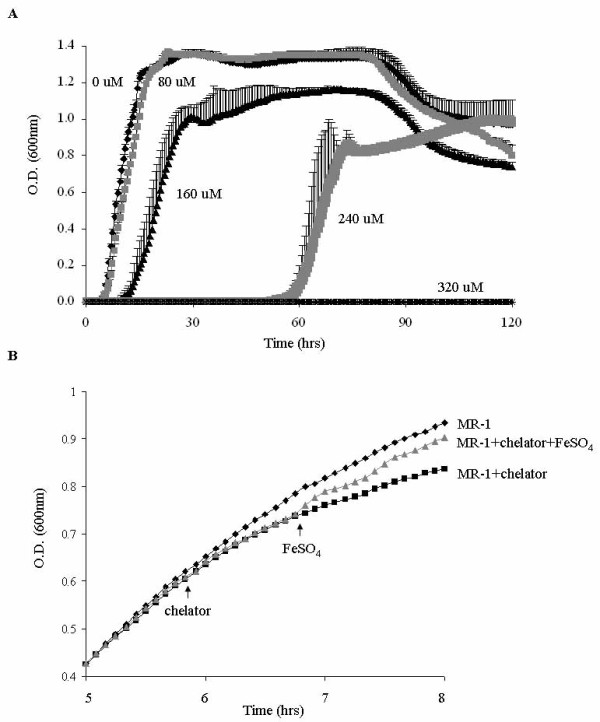
**(A) Growth curves of *S. oneidensis *MR-1 in liquid LB medium with different concentrations of iron chelator (2,2'-dipyridyl)**. Triplicates of cells grown to mid-log phase were diluted 1:100 in fresh LB liquid medium, and cell density was measured every half hour for five consecutive days. The standard deviations are shown as error bars. (B) When MR-1 cultures were grown to mid-log phase (O.D._600 nm _= 0.6), 160 μM 2,2'-dipyridyl was added to inhibit bacterial growth, which was subsequently reversed by repleting iron with 200 μM FeSO_4_. The arrows represents the time when 2,2'-dipyridyl or iron was added. Data are averages for triplicate cultures.

To examine the iron response of *S. oneidensis*, the wild-type strain MR-1 was grown in LB medium with different concentrations of the iron chelator 2,2'-dipyridyl used to deplete iron. LB medium, a rich source of iron (~17 μM) [26], was used to provide sufficient biomass for the following microarray experiments. Iron depletion imposed a challenge to cell proliferation and survival, resulting in an extended lag phase, slower growth rate at mid-log phase, and lower cell density at stationary phase (Fig. 1A). While 80 μM 2,2'-dipyridyl had a marginal effect on the growth rate of MR-1, the cells displayed clear growth inhibition with 160 μM and 240 μM 2,2'-dipyridyl. When 320 μM 2,2'-dipyridyl was used, cell growth was completely arrested throughout five days of observation.

To test whether iron depletion led to a rapid physiological change, MR-1 was grown to mid-log phase (OD_600 _= 0.6), then 2,2'-dipyridyl was added to a final concentration of 160 μM, followed by addition of ferrous sulfate after one hour. Iron depletion rapidly slowed growth within an hour of adding 2,2'-dipyridyl. Growth was recovered by the subsequent addition of ferrous sulfate (Fig. 1B).

### Transcriptomics and gene co-expression network

For microarray experiments, MR-1 cultures at mid-log phase were treated with 2,2'-dipyridyl. After one hour, iron was repleted with the addition of ferrous sulfate to the medium. 160 μM 2,2'-dipyridyl was used for iron depletion for the microarray experiments because it inhibited, but did not abrogate, cell growth (Fig. 1). Samples were collected at multiple time points during iron depletion and repletion and were used for global transcriptomic analyses. The reliability of the microarray data was validated by quantitative RT-PCR of selected genes (Table 1). A high correlation coefficient of 0.98 was observed between RT-PCR and the microarray results.

**Table 1:** Comparison of expression measurements by microarray and qPCR assays^a^

**GeneID**	**Methods**	**C5'**	**C20'**	**C60'**	**F5'**	**F20'**	**F60'**
TonB1	Microarray	45.17	34.35	64.58	0.03	0.02	0.01
	
	qPCR	314.44	232.51	465.56	0.02	0.007	

ExbB1	Microarray	37.79	56.59	56.36	0.05	0.03	
	
	qPCR	566.49	2203.76	813.80	0.014	0.001	

ExbD1	Microarray	7.33	13.87	9.45	0.21	0.16	
	
	qPCR	133.01	164.31	52.18	0.37	0.03	

SO3032	Microarray	1.80	9.20	19.42	--	0.14	
	
	qPCR	6.87	197.48	1391.45	1.05	0.008	

SdhA	Microarray	0.75	2.23	1.86	0.76	0.89	
	
	qPCR	0.37	3.55	2.06	1.16	1.65	

AcnA	Microarray	0.99	2.23	8.22	0.86	0.21	
	
	qPCR	1.27	3.25	66.43	2.01	0.06	

^a^Values greater than 1 indicate up-regulation and values less than 1 indicate down-regulation. A correlation coefficient of 0.98 was calculated after converting the RT-PCR and microarray data in the table to their logarithmic values. --: not detected.

**Figure 2:**
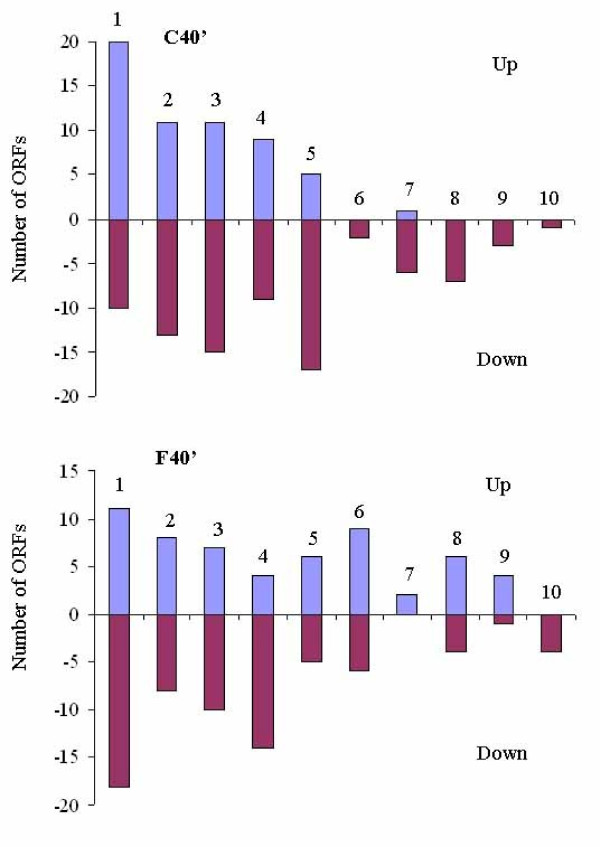
**Up- or down-expressed genes grouped by functional categories after 40 minutes of iron depletion (C40') and repletion (F40')**. The following functional categories are indicated as: 1. Transport and binding proteins; 2. Regulatory functions; 3. Energy metabolism; 4. Adaptation to atypical conditions; 5. Biosynthesis of cofactors; 6. Protein synthesis; 7. Central intermediary metabolism; 8. DNA turnover; 9. Cell envelope; and 10. Chemotaxis and motility.

The number of genes showing significant differential gene expression at each time point is listed in Table 2. The identification of 21 genes induced within one minute of iron depletion, many of which encode iron acquisition systems, clearly indicates that iron response is a very rapid process in *S. oneidensis*. Also notable is the fact that the total numbers of differentially expressed genes are smaller at earlier time points of iron depletion and repletion than those at later time points, at which other effects (such as accumulation of intermediate products and change in growth rate) may impact gene expression profiles. As is common in microarray datasets, a large portion of the up- or down-regulated genes corresponds to genes with unknown function, indicating a much broader iron stimulon and regulon than have been deduced solely on the basis of gene annotation. When the genes with significant changes were classified according to their functional categories, several groups were predominantly represented: transport and binding proteins (mostly iron acquisition systems), regulatory functions, energy metabolism, adaptation to atypical conditions, and biosynthesis of cofactors or proteins, as exemplified at 40 minutes of iron depletion and repletion (Fig. 2).

**Table 2:** The number of genes regulated at each time point

**Time points**	**C1'**	**C5'**	**C10'**	**C20'**	**C40'**	**C60'**	**F1'**	**F5'**	**F10'**	**F20'**	**F40'**	**F60'**
Up-regulated genes	21	35	43	68	100	96	6	46	51	120	82	152

Down-regulated genes	3	7	21	152	177	152	7	25	45	144	110	143

To gain more insights into the iron-responsive genes, a gene co-expression network containing a total of 128 genes partitioned into twelve modules (Additional file 1) was constructed (see Methods for details). Further analyses indicated that each module contains a functionally coherent set of genes. Large modules of genes related to iron acquisition, anaerobic energy metabolism and protein degradation are the most predominant in the network, suggesting that they are the major iron-responsive biological pathways in *S. oneidensis*. In addition, gene modules of DNA metabolism, sulfur metabolism, protein synthesis and aerobic energy metabolism also were identified.

### Module of iron acquisition systems

**Figure 3:**
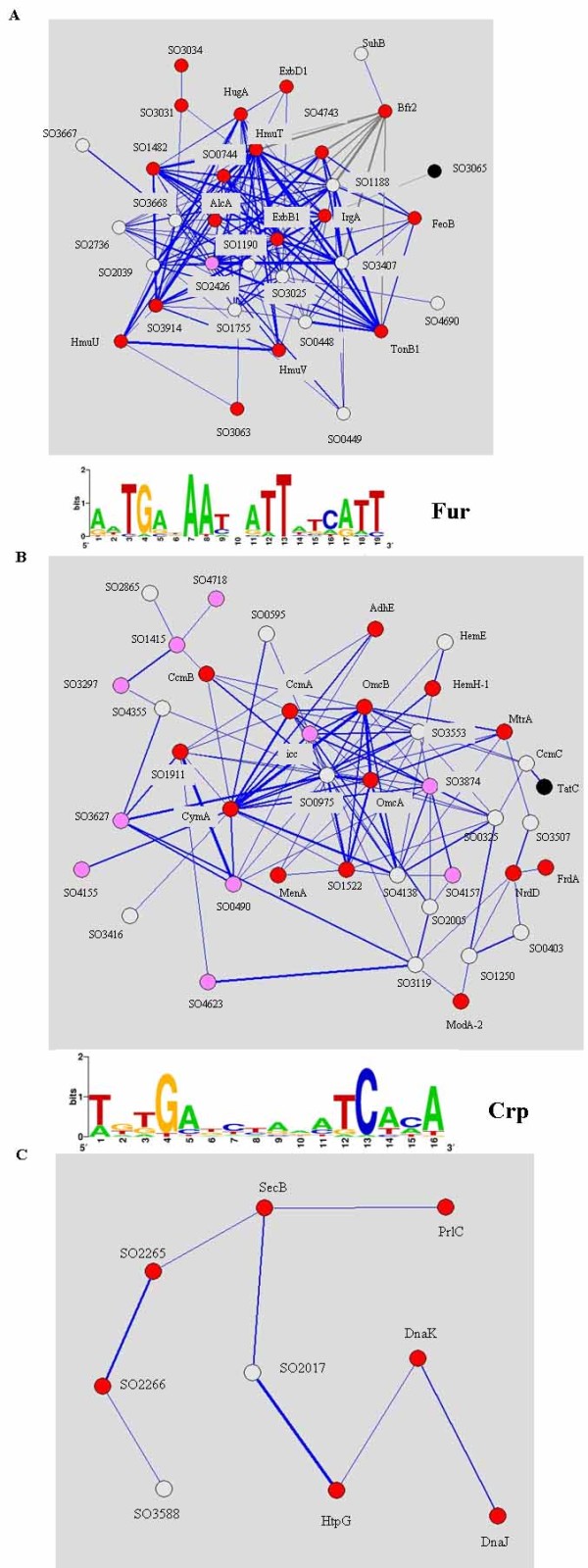
**(A) Module of iron acquisition systems**. Each node represents a gene and the width of the line represents the correlation coefficient of two linked genes. Blue and gray lines indicate positive and negative correlation coefficients, respectively. Colors are assigned to nodes according to their functional categories: red represents known iron acquisition systems, lavender represents transcriptional regulator, white represents unknown genes, and the black node depicts a gene whose association to other genes is not yet understood. The sequence logo of the consensus sequence in the promoter regions of genes in the module of iron acquisition systems was generated by the Gibbs Recursive Sampler program [28] and Weblogo program [50]. (B) Module of genes related to anaerobic energy metabolism. Colors are assigned as described in Fig. 3A except that red nodes represent genes with known roles in anaerobic energy metabolism. (C) Module of genes involved in protein degradation. Colors are assigned to nodes according to their functional categories: red represents genes that function in protein degradation, and white represents genes with unknown function.

The large module shown in Fig. 3A is composed almost exclusively of iron acquisition systems, suggesting that these genes are highly co-regulated under iron depletion and repletion conditions. These genes include the TonB system (TonB1-ExbB1-ExbD1), the hemin ABC transporter (HmuT, HmuU and HmuV), the siderophore synthetic protein (AlcA), and an iron-regulated outer membrane protein (IrgA). A majority of genes were strongly induced by iron depletion and repressed by iron repletion (see Table 3 for details). Notably, two homologous TonB systems located at the inner membrane have been annotated in *S. oneidensis*. The TonB1-exbB1-exbD1 operon was induced 2.4–64.6 fold during iron depletion and then repressed up to 100 fold by iron repletion, while TonB2-exbB2-exbD2 was not regulated by iron. Consistently, a "Fur box" was identified upstream of the TonB1-exbB1-exbD1 operon, but not upstream of the TonB2-exbB2-exbD2 operon [3]. In contrast, three genes (Bfr2, SO3065 and SuhB) display expression patterns opposite to the iron acquisition systems (Fig. 3A). The presence of Bfr2 can be explained as a bacterioferritin subunit that is regulated in the opposite way to iron acquisition.

**Table 3:** Three major modules in gene co-expression network^a^

Gene	Annotation	Change in expression at:											
		
		C1'	C5'	C10'	C20'	C40'	C60'	F1'	F5'	F10'	F20'	F40'	F60'
**Iron acquisition**													
SO0448	conserved hypothetical inner membrane protein	3.40	--	--	5.00	3.41	4.30	0.56	0.46	0.26	0.21	0.20	0.15
SO0449	conserved iron-regulated membrane protein	4.00	4.61	3.88	3.06	3.76	3.20	1.07	0.67	0.49	0.59	0.48	0.35
SO0744	iron(III) ABC transporter, periplasmic iron(III)-binding protein	2.92	3.42	3.72	3.61	5.40	6.93	0.50	0.16	0.12	0.12	0.20	0.17
SO1111	bacterioferritin subunit 2 (Bfr2)	0.53	0.45	0.41	0.36	0.41	0.56	2.56	3.41	6.00	--	5.75	5.61
SO1188	unknown inner membrane protein	8.66	9.94	10.62	14.47	10.91	8.38	0.47	0.12	0.09	0.11	0.24	0.10
SO1190	ABC-type Co2+ transporter, a periplasmic component	1.06	2.57	4.59	--	6.28	3.70	0.90	0.52	0.18	0.07	0.08	0.07
SO1482	TonB-dependent receptor	3.50	3.44	6.62	7.12	8.44	8.13	0.62	0.21	0.05	0.06	0.03	0.03
SO1755	phosphoglucomutase/phosphomannomutase family protein	1.37	1.63	2.09	2.77	3.31	4.19	0.55	0.38	0.27	0.21	0.26	0.21
SO1784	ferrous iron transport protein B (FeoB)	3.74	4.98	4.07	2.45	2.49	2.29	0.62	0.25	0.20	0.13	0.23	0.14
SO2039	signalling protein with EAL domain	3.09	3.89	3.39	4.86	4.81	--	--	--	--	--	--	--
SO2260	extragenic suppressor protein (SuhB)	0.59	0.49	0.43		0.41	0.45	2.52	1.78	2.49	6.21	3.63	5.58
SO2426	two component transcriptional regulator	11.65	13.67	10.06	7.88	8.90	10.00	0.57	0.11	0.07	0.06	0.05	0.06
SO2736	conserved hypothetical outer membrane protein	3.88	5.33	4.47	4.37	4.78	5.23	0.56	0.33	0.40	0.27	0.28	0.25
SO3025	unknown protein	1.85	2.54	3.71	5.24	6.75	6.11	0.71	0.28	0.25	0.23	0.23	0.16
SO3030	alcaligin biosynthesis enzyme (AlcA)	6.24	8.34	14.64	12.87	23.64	12.45	1.90	0.75	0.80	0.18	0.12	0.11
SO3032	alcaligin biosynthesis protein (AlcC)	1.65	1.80	3.94	9.20	21.09	19.42	1.23	--	0.72	0.14	0.42	--
SO3034	ferric iron reductase protein (AlcE)	1.07	1.02	1.60	3.76	5.51	5.25	0.65	1.01	0.50	0.15	0.18	0.11
SO3063	sodium:alanine symporter family protein	1.04	1.67	2.11	4.10	4.38	4.68	0.85	0.54	0.23	0.41	0.54	0.29
SO3065	colicin V production protein	--	--	0.33	0.35	0.37	0.42	2.16	--	1.29	2.73	2.93	3.30
SO3407	conserved iron-regulated membrane protein	11.36	17.13	18.98	18.04	13.31	10.14	0.68	0.10	0.05	0.06	0.13	0.04
SO3667	heme iron utilization protein	1.22	2.01	6.03	--	17.15	13.53	0.83	0.65	0.33	0.08	0.02	0.01
SO3668	HugX family protein	1.93	3.45	9.15	12.84	20.27	18.24	0.82	0.39	0.21	0.10	0.04	--
SO3669	heme transport protein (HugA)	19.42	41.21	56.72	--	74.82	69.78	0.44	0.07	0.03	0.04	0.04	--
SO3670	TonB1 protein	22.48	45.17	62.47	34.35	41.42	64.58	0.10	0.04	0.02	0.02	0.08	0.01
SO3671	TonB system transport protein (ExbB1)	16.33	37.79	50.11	56.59	51.37	56.36	0.59	0.05	0.02	0.03	0.03	--
SO3672	TonB system transport protein (ExbD1)	2.43	7.33	11.09	13.87	10.34	9.45	0.84	0.21	--	0.16	0.32	--
SO3673	hemin ABC transporter (HmuT)	2.20	7.30	13.52	26.71	17.87	19.86	0.91	0.33	0.07	0.06	0.11	0.04
SO3674	hemin ABC transporter, permease protein (HmuU)	1.08	2.53	6.74	11.84	10.57	9.13	1.07	0.49	0.09	0.15	0.47	--
SO3675	hemin ABC transporter, ATP-binding protein (HmuV)	--	4.83	16.36	27.84	21.42	19.39	1.23	0.64	--	0.11	0.41	--
SO3914	TonB-dependent receptor (iron uptake)	2.89	4.45	7.32	--	9.38	11.30	0.74	0.24	0.14	0.05	0.15	0.03
SO4523	iron-regulated outer membrane protein (IrgA)	3.53	5.23	9.19	14.16	23.22	25.92	0.54	0.21	0.08	0.04	0.13	0.04
SO4690	dolichyl-phosphate-mannose-protein mannosyltransferase family protein	1.46	1.45	1.62	2.09	2.16	2.02	1.02	0.71	0.51	0.64	0.48	0.56
SO4743	TonB-dependent receptor	8.12	9.18	7.07	4.48	4.29	3.23	0.66	0.18	0.09	0.08	0.19	0.13
													
**Anaerobic energy metabolism**													
SO0261	heme exporter protein (CcmC)	0.85	--	--	0.26	0.23	0.21	1.24	2.05	1.53	2.81	2.49	2.57
SO0262	heme exporter protein (CcmB)	1.76	1.38	0.79	0.21	0.23	--	--	--	--	--	--	--
SO0263	heme exporter protein (CcmA)	1.01	0.73	0.59	0.12	0.14	0.15	1.45	2.15	2.17	2.63	2.49	3.13
SO0325	dsrE-related protein	0.66	0.56	0.67	0.12	0.08	0.09	1.56	3.36	2.41	2.59	2.64	2.23
SO0398	FAD-binding subunit of inner membrane respiratory complex (FrdA)	1.72	1.74	1.04	0.24	0.23	0.20	0.96	2.14	1.99	2.55	2.03	2.61
SO0403	expressed protein	--	--	--	0.11	0.09	0.12	1.35	2.16	2.46	0.91	1.63	1.27
SO0435	uroporphyrinogen decarboxylase (HemE)	1.27	1.46	0.95	0.32	0.19	0.15	1.50	2.33	2.72	3.64	5.01	5.42
SO0490	transcriptional regulator	--	0.89	0.78	0.08	0.10	--	--	--	--	--	--	--
SO0595	expressed protein	1.04	0.97	0.92	--	0.09	--	--	--	--	--	--	--
SO0975	conserved hypothetical inner membrane protein	1.07	1.15	0.58	0.06	0.06	0.05	1.09	2.02	1.80	4.74	3.06	3.56
SO1250	conserved hypothetical protein	1.02	1.01	0.53	0.15	0.14	0.15	1.20	2.54	3.09	2.14	1.61	1.32
SO1415	transcriptional regulator, TetR family	1.05	0.88	0.57	0.04	0.05	--	--	--	--	--	--	--
SO1522	L-lactate permease, integral IM protein	1.08	0.95	0.70	0.18	0.24	0.15	2.13	3.43	3.33	5.55	4.03	4.27
SO1777	periplasmic decaheme cytochrome c, Fe(III) and Mn(IV) reduction (MtrA)	0.97	--	--	0.11	0.08	0.11	1.64	3.47	4.26	3.19	3.99	5.14
SO1778	decaheme cytochrome c (MtrC)	0.97	1.26	0.69	0.07	0.08	0.06	2.44	6.04	3.47	4.27	3.93	7.85
SO1779	Outer membrane decaheme cytochrome c (OmcA)	1.65	1.93	0.94	0.09	0.07	0.06	2.10	7.04	6.76	8.51	6.91	10.11
SO1910	1,4-dihydroxy-2-naphthoate octaprenyltransferase (MenA)	1.24	1.04	0.71	0.29	0.28	0.22	1.41	2.21	2.22	3.21	2.87	3.29
SO1911	oxidoreductase, short chain dehydrogenase/reductase family	0.71	0.66	0.47	0.03	0.06	0.04	1.90	3.72	2.60	5.44	3.71	3.01
SO2005	dksA-type zinc finger protein	0.46	0.47	0.19	0.05	0.07	--	--	--	--	--	--	--
SO2019	ferrochelatase (HemH 1)	1.33	0.94	0.82	0.18	0.18	0.16	1.29	2.53	2.14	4.77	3.84	5.56
SO2136	aldehyde-alcohol dehydrogenase (AdhE)	0.62	0.41	0.43	0.09	0.11	0.08	2.29	3.05	3.05	7.13	3.75	5.88
SO2834	anaerobic ribonucleoside-triphosphate reductase (NrdD)	1.16	1.83	0.47	0.12	0.09	0.12	0.80	2.27	2.19	2.12	1.55	1.24
SO2865	L-lysine exporter, putative	1.66	1.31	0.70	0.16	0.30	--	--	--	--	--	--	--
SO3119	conserved hypothetical protein	--	--	--	0.19	0.20	0.26	2.18	5.48	2.87	2.11	1.70	2.00
SO3297	transcriptional regulator, LysR family	1.24	0.98	0.72	0.16	0.21	0.29	1.09	0.92	1.08	1.50	1.57	--
SO3416	hypothetical protein	1.13	1.27	0.95	0.24	0.25	0.38	1.77	1.89	2.25	1.05	1.98	1.37
SO3507	conserved hypothetical protein	2.08	1.70	1.45	0.19	0.17	0.10	1.73	--	8.41	3.10	3.00	2.60
SO3553	sulfate permease family protein	0.68	0.74	0.67	0.19	0.17	0.11	2.24	3.94	3.60	7.45	5.47	6.92
SO3627	transcriptional regulator, TetR family	1.17	1.07	0.72	0.03	0.06	0.06	0.92	6.40	3.07	3.19	2.99	1.74
SO3874	transcriptional regulator, LysR family	0.60	0.48	0.30	0.07	0.05	0.05	1.72	4.56	3.75	3.81	2.79	2.96
SO3901	lacZ expression regulator (icc)	1.15	0.75	0.60	0.21	0.20	0.15	1.71	3.61	2.74	3.24	2.23	2.74
SO4138	expressed periplasmic protein	0.95	0.96	0.68	0.13	0.09	0.11	1.42	4.10	2.55	3.76	3.46	3.63
SO4155	sensor histidine kinase for thiosulfate/tetrathionate response (TtrS)	0.72	0.65	0.72	0.10	--	--	--	--	--	--	--	--
SO4157	two component transciptional regulator for thiosulfate/tetrathionate response (TtrR)	0.39	0.66	--	0.12	--	0.09	1.66	2.78	--	1.98	2.02	2.60
SO4204	Sec-independent periplasmic protein translocation protein (TatC)	0.87	0.64	0.61	--	0.27	0.25	1.64	--	1.72	2.53	2.95	3.58
SO4355	cAMP-binding protein	2.73	2.02	1.62	0.19	0.26	0.22	2.01	2.24	1.33	2.44	1.86	2.69
SO4448	molybdenum ABC transporter, periplasmic molybdenum-binding protein (ModA-2)	1.65	1.84	1.00	0.33	0.33	0.43	1.17	1.39	1.28	1.35	1.18	1.06
SO4591	tetraheme cytochrome c (CymA)	--	--	--	0.17	0.15	0.11	2.83	7.01	3.75	3.76	5.27	7.71
SO4623	two component transcriptional regulator	0.68	0.51	0.44	0.11	0.12	0.14	1.21	1.69	1.30	1.31	1.46	1.39
SO4718	two component Sigma54 specific transcriptional regulator for tungstate (molybdate) transport	1.79	1.69	1.42	0.42	0.40	0.47	1.30	1.86	1.83	2.25	2.38	2.82
													
**Protein degradation**													
SO0052	protein export chaperone (SecB)	0.93	1.00	0.68	0.66	2.44	--	--	--	--	--	--	--
SO1126	chaperone protein (DnaK)	0.80	1.14	0.84	0.68	1.79	1.60	0.76	0.95	0.41	0.05	0.06	0.04
SO1127	chaperone protein (DnaJ)	1.19	1.07	0.73	1.00	1.67	1.11	1.22	1.29	0.76	0.23	0.17	0.24
SO2016	heat shock protein (HtpG)	0.88	0.82	0.58	1.15	2.53	1.74	0.93	1.05	0.71	0.10	0.22	0.06
SO2017	Unknown Protein	0.83	0.81	0.56	1.07	2.78	1.64	1.19	0.91	0.61	0.16	0.20	0.14
SO2265	scaffold protein for Fe-S cluster assembly (IscU)	--	1.48	1.33	1.45	4.66	3.74	0.82	0.59	0.36	0.09	0.25	0.19
SO2266	iron recruitment protein (IscA)	1.18	1.28	1.05	1.45	4.46	4.68	1.04	0.81	0.23	0.12	0.25	0.23
SO3588	gpr1/fun34/yaaH family protein	1.09	1.08	1.02	1.39	1.98	2.88	1.20	0.61	0.38	0.44	0.57	0.76
SO4699	oligopeptidase A (PrlC)	0.98	0.92	0.75	1.49	2.36	1.61	1.05	1.08	0.44	0.13	0.28	0.12

Expression ratio values greater than 1 denote increases in gene expression and values between 0 and 1 indicate decreases in gene expression. --: not detected.

Five genes (SO0448, SO0449, SO1188, SO2736 and SO3407) encoding unknown conserved inner or outer membrane proteins are regulated by iron, raising the possibility of novel iron acquisition systems. In addition, a single transcriptional regulator (SO2426) clusters with iron acquisition systems. Very recently, an independent genetic study showed that SO2426 regulated siderophore production, confirming its role in regulating iron acquisition [27].

To identify the possible transcriptional regulator for genes in this module, their upstream intergenic regions were searched for common motifs using the Gibbs Recursive Sampler [28]. A motif almost identical to the predicted "Fur box" in *S. oneidensis *[3] was identified (Fig. 3A). Thus, the genes in this module appeared to be directly controlled by Fur. Consistently, most of these genes were de-repressed in a *fur *deletion mutant [3,25].

### Module of anaerobic energy metabolism

In general, genes in this module are repressed by iron depletion and induced by repletion (see Table 3 for details). This module contains many genes contributing to anaerobic energy metabolism, including *c*-type cytochromes, which function in anaerobic metal reduction (e.g., CymA, MtrA, MtrC and OmcA); anaerobic ribonucleoside-triphosphate reductase (NrdD); the FAD-binding subunit of the inner membrane respiratory complex (FrdA); alcohol dehydrogenase (AdhE); biosynthetic genes of cytochromes such as the heme exporter (HemE and HemH-1); and cytochrome maturation systems (CcmA, CcmB and CcmC) (Fig. 3B). In addition, ten transcriptional factors are clustered with the functional genes, implicating that they function to regulate this process. Although none of them has yet been experimentally characterized, we note that SO0490, SO1415, SO3297, SO3627, SO3874, SO4155, SO4157, SO4623 and SO4718 are induced 2.8–8.0 fold in a microarray experiment of *S. oneidensis *MR-1 under anaerobic, iron-reducing conditions as compared to O_2_-reducing conditions (Yang *et al*., unpublished data). In addition, SO0490 also is induced 4 fold under anaerobic uranium-reducing conditions [29].

The Gibbs Recursive Sampler was used to identify the consensus sequence motif in the promoter regions of genes in the module. The search resulted in a palindromic sequence of "**T**G**TGA**TCTANA**TCA**C**A**", which was almost identical to the core binding motif of the global transcriptional factor CRP (**TGTGA**TCTAGA**TCACA **in *E. coli*) [30]. In *S. oneidensis*, *crp *mutants were deficient in reducing Fe(III), Mn(IV), nitrate, fumarate and DMSO [31]. The identification of a conserved CRP-binding site at the promoters of genes in this module suggests that inactivation of Crp could lead to repression of multiple branches of the anaerobic energy metabolism pathway and thus to deficiency in reducing electron acceptors.

### Module of protein degradation

A module of protein degradation was identified in the network (Fig. 3C). These genes generally are induced by iron depletion and repressed by iron repletion (Table 3). They include heat shock proteins (DnaK, DnaJ and HtpG) and heat shock protein 70 (Hsp70) system-interacting proteins (IscU and IscA) [32]. An RpoH (σ^32^) binding site (CTTGAAA and CCCCAT) was identified -35 and -10 upstream of genes in this module, which was consistent with previous observations in *E. coli *that RpoH was responsible for regulation of heat shock proteins [33]

### Other biological processes

Other iron-responsive biological processes are related to DNA metabolism, sulfur metabolism, protein synthesis and aerobic energy metabolism. A number of genes encoding ribosomal proteins (e.g. L31, L9, L23, L6 and S7) are induced by iron repletion (Table 4), which correlates well with the rapid resumption of bacterial growth after iron addition. Additionally, genes related to aerobic energy metabolism are induced by iron depletion and repressed by iron repletion (Table 4), possibly implying that the energy required for coping with iron depletion is greater than that required for normal growth. These genes include methylcitrate synthase (PrpC), 2-methylisocitrate lyase (PrpB), 2-methyl citrate dehydratase (AcnD), isocitrate dehydrogenase (SO1538), and malate dehydrogenase (SfcA).

**Table 4:** Other selected genes differentially expressed during iron depletion and repletion^a^

Gene	Annotation	Change in expression at:											
		
		C1'	C5'	C10'	C20'	C40'	C60'	F1'	F5'	F10'	F20'	F40'	F60'
**Protein synthesis**													
SO0227	ribosomal protein S7 (RpsG)	1.08	1.14	1.09	--	0.73	0.62	1.33	--	2.58	1.93	2.37	2.37
SO0233	ribosomal protein L23 (RplW)	--	--	--	--	0.61	0.55	1.84	--	3.42	3.64	3.78	--
SO0246	ribosomal protein L6 (RplF)	0.94	0.93	0.79	1.08	0.78	0.72	1.22	1.77	2.04	--	2.83	2.87
SO1205	ribosome-binding factor A (RbfA)	1.13	0.88	0.65	0.95	0.60	0.48	1.23	1.09	1.40	2.65	3.34	3.56
SO3927	ribosomal protein L9 (RplI)	--	--	--	1.33	0.50	0.69	1.29	--	--	3.58	3.56	3.83
SO4120	ribosomal protein L31(RpmE)	0.39	0.35	0.36	0.29	0.44	0.26	2.68	2.51	3.68	6.47	4.22	6.00
													
**Aerobic energy metabolism**													
SO0343	2-methyl citrate dehydratase (AcnD)	1.19	0.99	0.86	2.23	3.53	8.22	1.14	0.86	0.47	0.21	0.25	0.34
SO0344	methylcitrate synthase (PrpC)	0.81	0.61	0.52	1.77	4.17	8.74	0.90	0.63	0.30	0.11	0.19	0.22
SO0345	2-methylisocitrate lyase (PrpB)	0.73	0.70	0.71	2.54	4.35	8.07	1.05	0.45	0.24	0.15	0.33	0.33
SO1538	isocitrate dehydrogenase	0.69	0.95	1.01	5.25	4.04	4.08	0.73	0.44	0.62	0.55	0.66	0.59
SO3855	malate dehydrogenase (SfcA)	0.88	0.92	1.14	3.16	2.73	2.48	1.03	0.97	1.11	1.30	1.17	1.37

^a^Expression ratio values greater than 1 denote increases in gene expression and values between 0 and 1 indicate decreases in gene expression. --: not detected.

### Mutant analyses

**Figure 4:**
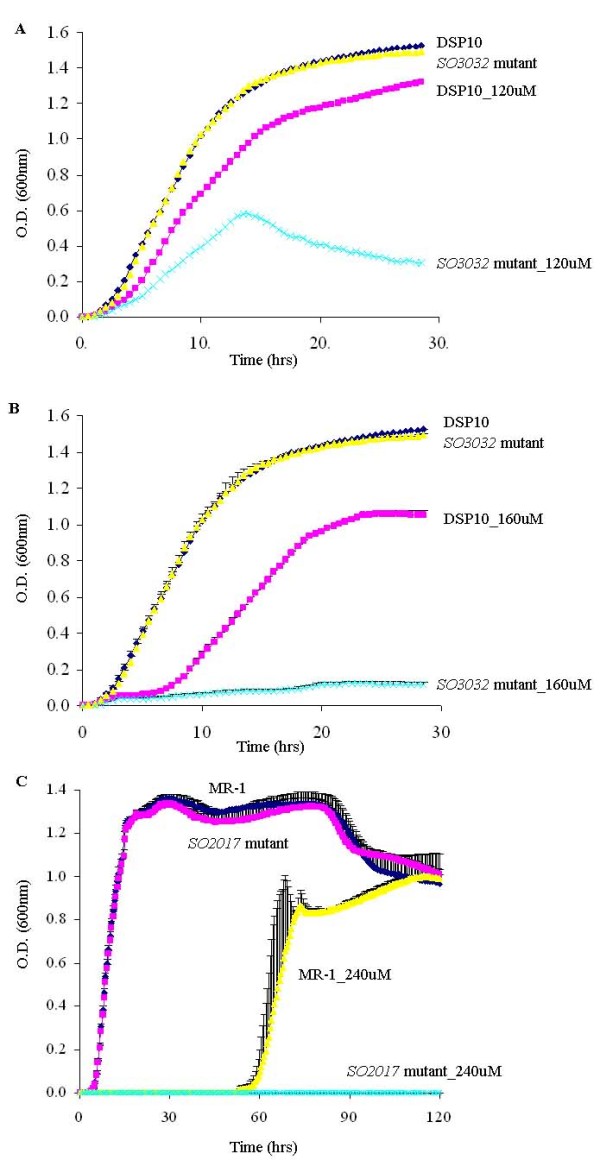
**(A) Growth of *SO3032 *mutant and its parent strain DSP10 were compared in the presence of 120 μM 2,2'-dipyridyl**. Cell density was measured at OD_600 _every 30 min for five days. Data are averages for triplicate cultures. (B) Growth of *SO3032 *mutant and DSP10 were compared in the presence of 160 μM 2,2'-dipyridyl. (C) Experimental verification of the involvement of SO2017 in iron response. Both strains were grown to OD_600 _of 0.6 before transferring to fresh LB medium in 1:100 dilutions with or without 240 μM 2,2'-dipyridyl.

To test the importance of iron acquisition genes under iron depleted conditions, a putative alcaligin biosynthesis protein SO3032 was selected for examination. A mutant of SO3032 and its parental strain DSP10 were compared under iron depleted conditions. The mutant displayed a severe growth defect when grown in the presence of 120 μM iron chelator (Fig. 4A), and had only marginal growth when grown in the presence of 160 μM iron chelator (Fig. 4B). This suggests that SO3032, which is induced ~20 fold after 40 minutes of iron depletion, is required for surviving iron starvation.

To determine the significance of a protein degradation module for iron depletion, a gene encoding a hypothetical protein (SO2017) was selected for mutagenesis. This gene is located in the same operon as the heat shock protein (HtpG), and its amino terminus contains a thioredoxin domain that might participate in redox reactions. Recently, it was shown to be required for bacterial survival of heat shock [34]. Although both the *SO2017 *mutant and its parental strain MR-1 grew almost indistinguishably in the absence of iron chelator 2,2'-dipyridyl, the mutant failed to grow under iron depleted conditions after incubating for five days at 30°C (Fig. 4C). Therefore, lack of SO2017 prohibited the cells' ability to survive iron depletion.

**Figure 5:**
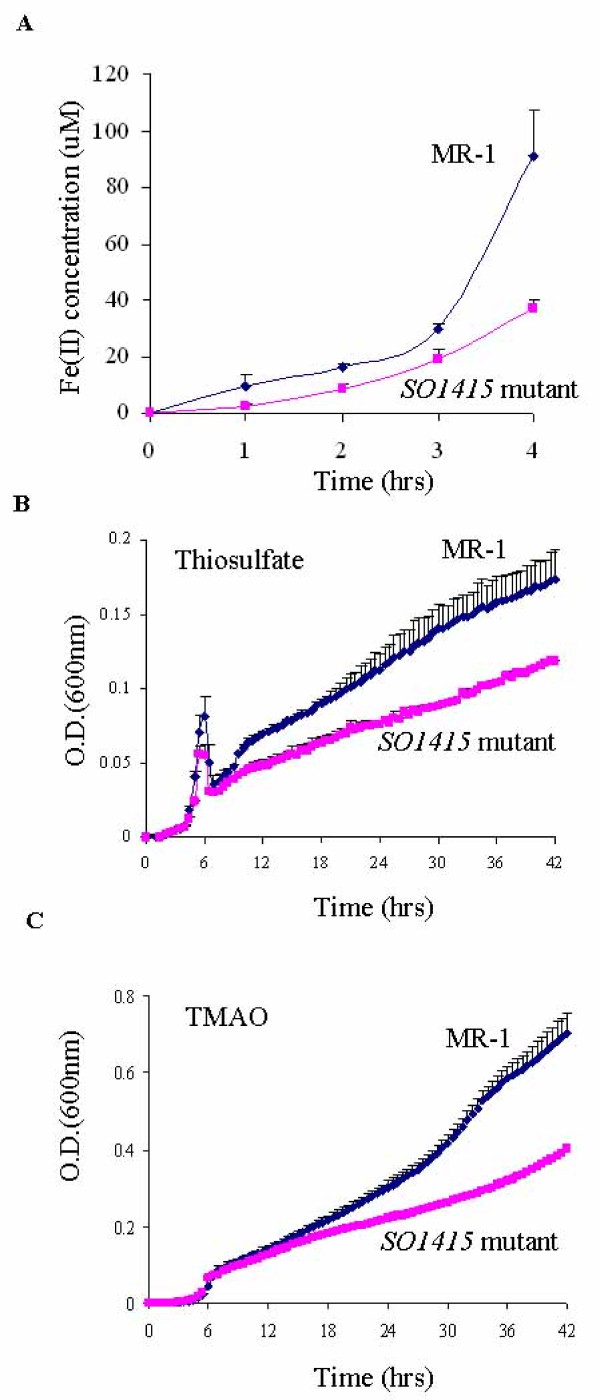
**(A) Reduction of Fe(III) citrate by MR-1 and *SO1415 *mutant**. (B) Anaerobic growth curves of MR-1 and *SO1415 *mutant with thiosulfate as the electron acceptor. (C) Anaerobic growth curves of MR-1 and *SO1415 *mutant with TMAO as the electron acceptor.

Network analysis suggests that ten novel transcriptional factors might be involved in anaerobic energy metabolism. Among them, one gene (SO1415) was successfully inactivated from the MR-1 genome. The Fe(III) reduction rates of the *SO1415 *mutant and MR-1 were assessed by a ferrozine assay as described [35]. Less than half the amount of Fe(II) was produced in the mutant as compared to MR-1 after four hours of incubation (Fig. 5A), indicating that disruption of SO1415 impaired anaerobic Fe(III) reduction.

We also assessed possible growth deficiency of the *SO1415 *mutant under anaerobic conditions with 10 mM lactate as the electron donor, and one of the following non-metal electron acceptors: 10 mM thiosulfate, 3 mM fumarate, 2 mM TMAO, 1 mM nitrate or 3 mM DMSO. Significant growth deficiency was observed when thiosulfate or TMAO was supplied (Fig. 5B and 5C). In contrast, only very mild growth deficiency was observed when DMSO, fumarate or nitrate was supplied, and no growth deficiency was detected under aerobic conditions (data not shown). Together, these results suggest that SO1415 is involved in regulating specific branches of anaerobic energy metabolism.

## Discussion

In this work we show that iron response in *S. oneidensis *is a rapid process at both molecular and physiological levels. The use of the membrane permeable iron chelator 2,2'-dipyridyl rapidly sequesters both intra- and extra-cellular iron [7]. As a result, when cells were sampled after one minute of iron depletion, functional and regulatory genes of iron acquisition systems (HugA, TonB1, ExbB1, AlcA, SO4743 and SO2426) were induced greater than six fold. These genes appear to be direct targets of Fur as evidenced by the presence of "Fur boxes" in their promoters. Notably, Fur has a weak affinity for Fe(II) to form a repression complex [36], allowing for a rapid and sensitive response to a change in iron concentration.

The identification of an anaerobic energy metabolism module for iron experiments conducted under aerobic conditions is intriguing. A straightforward explanation would be that gradually limiting oxygen induces an anaerobic response when bacteria are grown in shake flask cultures. However, we show here that *S. oneidensis *continues to grow and cell density continues to increase during iron depletion, a condition which should further limit the concentration of oxygen and thus up-regulate the expression of genes as an anaerobic response. The expression of genes in the anaerobic energy metabolism module does not fit this model. Furthermore, a number of genes involved in aerobic response are induced by iron depletion (Table 4), while genes involved in anaerobic response and not co-factored with Fe(II) are not induced by iron depletion. These genes include UbiC, chorismate lyase involved in the initial steps of ubiquinone biosynthesis; Ppc, phosphoenolpyruvate carboxylase synthesizing oxaloacetate; Pta and AceK, enzymes converting pyruvate to acetate during fermentation; GlpK, glycerol kinase synthesizing glycerol-3-phosphate; Tdh, threonine dehydrogenase involved in the supply of reducing potential; and glutamate decarboxylase (SO1769), whose *E. coli *counterpart is highly induced by anaerobiosis [37]. Therefore, an alternative explanation is that an anaerobic energy metabolism module may function as iron-storage proteins to release previously sequestrated iron in their protein products during iron depletion and thereby elevate the intracellular free iron pool. The use of non-essential, iron-cofactor proteins for iron homeostasis has been well documented in *E. coli *[10,15]. Notably, a microarray study in *E. coli *indicates that a large number of genes related to anaerobic energy metabolism (e.g., Hyb and Frd) function in iron storage [10]. Such a mechanism seems to be present in *S. oneidensis *as well.

Ten transcriptional regulators were grouped within the module of anaerobic energy metabolism, suggesting that they may be involved in this process. We were able to generate a mutant of one of those genes (SO1415) and to experimentally verify its role in anaerobic energy metabolism. Genome analyses reveal that *S. oneidensis *has a large repertoire of transcriptional regulators, e.g., 88 two-component regulatory system proteins that enable the organism to adapt to a diversity of environmental conditions [12]. Nevertheless, most of the transcriptional regulators remain unstudied. The grouping of transcriptional regulators in anaerobic energy metabolism is an exciting finding; understanding this process is crucial to the potential utilization of *Shewanella *species to remediate U.S. Department of Energy's uranium-contaminated sites. In this regard, the rest of the transcriptional factors identified for anaerobic energy metabolism besides SO1415 are worthy of further investigation.

Bioinformatics analyses suggest that genes in the anaerobic energy metabolism module may be directly regulated by Crp. In *E. coli*, Crp modulates different biological processes and responds to glucose levels as a global transcriptional factor [38]. However, in *S. oneidensis*, Crp plays a critical role in anaerobic energy metabolism [31]. The identification of a Crp-binding site in this module provides a reasonable explanation for the function of Crp in multiple branches of anaerobic energy metabolism.

Transcriptomics and genetic studies suggest that protein degradation is involved in iron response. Lack of iron as a protein cofactor may impair the stability of a number of proteins. Induction of heat shock proteins may be necessary to process denatured or misfolded proteins. Intriguingly, no oxidative stress genes, such as Fe-superoxide dismutase (SodB) and genes in the SOS pathway (*e.g*., RecA, RpoD, RpoH, LexA, SsB, UmuC and UmuD) [39], were induced when iron was repleted. This is surprising since the excess of external iron is expected to provoke oxidative stress. It is possible that the concentration of iron used in this study is not sufficient to induce oxidative stress, or that *S. oneidensis *had yet not responded to oxidative stress during the period of time examined. Another possibility is that *S. oneidensis *employs novel pathways for oxidative stress response.

In *E. coli*, the TCA enzymes SdhA and AcnA are controlled by the regulatory cascade of Fur and the small RNA RyhB. Consequently, they are repressed under iron-depleted conditions [10,15]. This is not observed in *S. oneidensis *(Table 1). Indeed, we found that the expression of SdhA and AcnA in *S. oneidensis *was regulated neither in the *fur *mutant [2,3,25], nor in a strain that over-expresses RyhB (Yang et al., unpublished results), suggesting that SdhA and AcnA are not regulated by Fur and RyhB in *S. oneidensis*.

The regulation of iron acquisition genes by Fur is affirmed both by the presence of "Fur boxes" in the promoters and the abolishment of gene expression in *fur *mutants [3,25]. This mechanism is well conserved among γ-proteobacteria [1,9]. In contrast, it appeared that the regulation of anaerobic energy metabolism and protein degradation modules was largely Fur-independent, as very similar sets of genes also were identified when a *fur *mutant was exposed to iron depletion and repletion conditions [25]. This implies that transcriptional regulators other than Fur are also essential for iron response. Indeed, Fur-independent regulation of gene expression by iron has been observed in *E. coli*, *V. cholera*, and *H. Pylori *[1,7,8]. Nevertheless, we could not completely rule out the possibility of an indirect effect of Fur on anaerobic energy metabolism and protein degradation. It was notable that an earlier study showed that Crp was differentially expressed in a *fur *mutant [3], despite the fact that there was no obvious "Fur box" upstream of the Crp ORF.

## Conclusion

This report provides a glimpse into the physiological and molecular events of iron response in *S. oneidensis*. In addition to the Fur-regulated iron acquisition systems, numerous other genes are affected by changes of iron concentration. It will be interesting to further determine the mechanism of their involvement and regulation in iron response in future studies.

## Methods

### Bacterial strains and plasmids

Wild-type *S. oneidensis *MR-1 was used for physiology and transcriptomic studies. To generate an *SO2017 *deletion mutant from MR-1, the majority of the ORF was removed using PCR amplification with the primers A1 (5'AGCCTGTGAGCTCACGGG), A2 (5'TGTTTAAACTTAGTGGATGGGGGTTAG ATCGAGGATATT), B1 (5'CCCATCCACTAAGTTTAAACAGTTTGGCAAACCAAT ATC) and B2 (5'ACAATCGAGCTCTGCGAT), and a second cross-over PCR amplification with A1 and B2 using the mixed amplified fragments as templates was performed. The resulting product was cloned into the suicide plasmid pDS3.0 [3] and transformed into the *E. coli *WM3064 strain prior to conjugal transfer into MR-1. Correct in-frame deletion was verified by DNA sequencing of the region surrounding the DNA recombinant site. To generate a SO1415 mutant from MR-1, an internal fragment of SO1415 was amplified by PCR using primers (5'-TCCTTCGGACTCCCTGT; 3'-CCATCAGGTTTGCTAAATGT) and cloned into the suicide plasmid pKNOCK-Km^r ^with *E. coli *WM3064 as the host. After introducing the plasmid into MR-1 by conjugation, PCR was employed to amplify the genomic region surrounding the SO1415 locus, and subsequent sequencing confirmed the insertional disruption of SO1415. SO3032 and its parental strain DSP10 were described previously [40].

### Physiological studies and ferrozine assay

MR-1 was grown to mid-log phase (OD_600 _= ~0.6) and diluted 1:100 into 300 μl fresh LB liquid medium. A Type FP-1100-C Bioscreen C machine (Thermo Labsystems) was used to measure growth every 30 min. All physiological studies were done in triplicates so that the average and standard deviation could be calculated. A range of concentrations of the iron chelator 2,2'-dipyridyl (80 μM, 160 μM, 240 μM, and 320 μM) was prepared in water. Iron was repleted with the addition of freshly prepared ferrous sulfate solution in water to the cell culture to attain a final concentration of 200 μM. Routine aerobic culturing of *S. oneidensis *and *E. coli *strains was performed in Luria-Bertani (LB) medium (pH 7.2) at 30°C and 37°C, respectively. The medium for anaerobic culturing was supplemented with 10 mM lactate as the electron donor and one of the following electron acceptors: 10 mM ferric citrate, 2.5 mM Mn(IV) dioxide, 0.1 mM K2CrO4, 10 mM fumarate, 20 mM TMAO, 10 mM DMSO, 1 mM nitrate, or 10 mM thiosulfate. For growth under anaerobic conditions, a Type FP-1100-C Bioscreen C machine placed in a Coy anaerobic chamber was used.

To measure the Fe(III) reduction rate, cells were grown anaerobically to mid-log phase in 10 ml fresh LB medium supplemented with 10 mM fumarate and 10 mM lactate. The cells were then spun down, washed with anaerobically prepared LB medium, and divided into aliquots of ~5 × 10^7 ^cells that were transferred into 5 ml LB medium supplemented with 10 mM lactate and 10 mM Fe(III) dioxide in a Coy anaerobic chamber. The ferrozine assay was performed as previously described [35]. Uninoculated medium containing 10 mM Fe(III) served as the abiotic control.

### RNA preparation

Four biological replicates of MR-1 were grown to the mid-log phase (OD_600 _= 0.6) in 100 ml LB medium in 500 ml shake flasks. 5 ml samples were collected at time 0, and then at 1, 5, 10, 20, 40, and 60 min after adding 2,2'-dipyridyl to attain a final concentration of 160 μM. Thereafter, ferrous sulfate was added, and cells were collected at 1, 5, 10, 20, 40, and 60 min. Cultures were vigorous shaked at 250–300 rpm to improve aeration. Cultures were sampled by centrifuging 6 ml cells at 14 krpm for one minute. After removing the supernatant, the cells were snap frozen in liquid nitrogen and stored at -80°C. Total RNA was extracted using Trizol Reagent (Invitrogen) as previously described [3]. RNA samples were treated with RNase-free DNase I (Ambion) to digest residual chromosomal DNA and then purified with RNeasy Kit (Qiagen) prior to spectrophotometric quantification at 260 and 280 nm.

### Microarray hybridization, scanning and quantification

To allow for comparison of any pairs of samples, MR-1 genomic DNA was used as a common reference in each microarray experiment. This strategy has been successfully employed in several studies [41-43]. In brief, cDNA was produced in a first-strand reverse transcription (RT) reaction with random hexamer primers (Invitrogen) and labeled with Cy5 dUTP (Amersham Biosciences) by direct labeling. *S. oneidensis *MR-1 genomic DNA (gDNA) was amplified by Klenow (Invitrogen), and Cy3 dUTP (Amersham Biosciences) was incorporated into the product. Fluorescein-labeled probes were purified using the Qiaquick PCR purification kit (Qiagen).

The *S. oneidensis *microarray contained a total of 4,761 PCR products, representing probes of ~99% of the predicted ORFs of the MR-1 genome [2,3,25]. Microarray slides were pre-hybridized at 50°C for about one hour to remove unbound DNA probes in a solution containing 50% (V/V) formamide, 9% H_2_0, 3.33% SSC (Ambion), 0.33% sodium dodecyl sulfate (Ambion), and 0.8 μg/μl bovine serum albuminin (BSA, New England Biolabs). Slides were hybridized at 50°C overnight with Cy5- and Cy3-labeled probes in the above solution, with 0.8% μg/μL herring sperm DNA (Invitrogen) replacing BSA, to prevent random binding. Pre-hybridization and hybridization steps occurred in hybridization chambers (Corning). Slides were washed on a shaker at room temperature as follows: 7 min in 1× SSC, 0.2% SDS; 7 min in 0.1× SSC, 0.2% SDS; and 40 s in 0.1× SSC.

A ScanArray Express Microarray Scanner (PerkinElmer) was used to scan the slides. Fluorescence and background intensity were quantified using ImaGene 6 software (BioDiscovery). All spots with signal-to-background ratios of less than 3 were discarded.

### Microarray data analysis

To calculate the ratios for different time points, samples collected during iron depletion (C1', C5', C10', C20', C40' and C60') were compared to the sample with no chelator (C0'). Samples collected during iron repletion (F1', F5', F10', F20', F40' and F60') were compared to C60'. (In our notation, 1' is 1 min, 5' is 5 min, etc.). Data analysis followed a standard published protocol [44]. A statistical model incorporating per-gene variance ("*z *values") was used to compute the posterior probability that the expression level of each gene changed in the direction indicated by the mean value. Ratios with |log_3_(ratio)| > 1 or |z| > 2.0 are considered significant.

Principles and details of Random Matrix Theory (RMT) based algorithms have been described in [34,45,46]. Application of RMT to large-scale biological data sets allows for grouping of functionally related genes and functional prediction of unknown genes. Compared to other network methods, RMT is unique in that it adopts two mathematical models to define the optimal threshold for removing microarray noise and hence produces an unsupervised network. The procedure to construct a gene co-expression network from the temporal gene expression profiles is as follows. The first step was to construct a gene expression correlation matrix M, whose elements are pair-wise Pearson correlation coefficients (*r*) of the experimental results. Next, a series of correlation matrices were derived from M using different cutoff values. If the absolute value of an element in M is less than the selected cutoff, the element is set to 0. Eigenvalues of the derived matrices were obtained by direct diagonalization of the matrix. Standard spectral unfolding techniques [47] were applied to have a constant density of eigenvalues and thus to determine the nearest neighbor spacing distribution P(s), which describes the fluctuation of eigenvalues of the correlation matrices.

We used the chi square test to determine two critical threshold values: r_l _at which P(s) starts to deviate from the Gaussian orthogonal ensemble at a confidence level of *p *= 0.001 and r_h _at which P(s) follows the Poisson distribution at a confidence level of *p *= 0.001. The critical point r_h _was used as the threshold for constructing the gene co-expression network. Applying the RMT method to the microarray data revealed a Pearson correlation coefficient of 0.91 as the minimum threshold to construct the gene co-expression network. Since gene co-expression networks are hierarchical [48], thresholds higher than 0.91 were used to identify additional functional modules, resulting in the twelve modules shown in file. The Pajek software [49] was used to visualize the gene co-expression network.

### Real-time RT-PCR

Real-time quantitative reverse transcription-PCR (RT-PCR) was performed as described previously [3], except that iQ SYBR green supermix (Bio-Rad) was used instead of SYBR green I. The sequences of primers used in this study are: TonB1 (5'-TCTAAACAGTCGCAGGAGC; 3'-TTGGTTGGCACTAACTCG); ExbB1 (5'-CTCC CCAAAAAAACAAGC; 3'-CAGTAAATCCTGCTGATGG); ExbD1 (5'-CAATATTATGG CGAGTTCACC; 3'-GTTAACTGCGCTTCAAACG); SO3032 (5'-CCATGAGAAGCTCAT CACACC; 3'-GCACGCGCTAAAGTAATACG); SdhA (5'-GAGCAGTTAAAAGCCATCC; 3'-GTTGTCCAATTCTAAACACTCG); AcnA (5'-ACCAACAAACGCTAGACTACC; 3'-ATCATCGCTCCACAAACC).

### Microarray data accession

The microarray data discussed in this publication have been deposited in NCBI's Gene Expression Omnibus and are accessible through GEO accession number GSE15334 .

## Authors' contributions

YY conceived and oversaw the study and wrote the manuscript. DPH, WX and ZY carried out the experiments. FL performed the RMT network reconstruction. LW constructed the microarray. MJ, PD and JJ performed statistical analyses and manuscript editing. APA, AVP and JZ coordinated the study and performed manuscript editing. All authors read and approved the final manuscript.

## Supplementary Material

Additional file 1 **Gene co-expression network inferred from the microarray data.** Each node represents a gene and the width of line represents the correlation coefficient of two linked genes. Blue and gray lines indicate positive and negative correlation coefficients, respectively. Colors are assigned to nodes according to their functional categories: red represents the major functional category of each cluster, as indicated by text; lavender represents transcriptional regulator; white represents unknown genes and black nodes are genes whose functional links to other genes are not yet understood. The italic bold numbers are the cutoffs used to isolate clusters. Click here for file
