# Supplementary figures and images for: Snapshot of iron response in Shewanella oneidensis by gene network reconstruction
Source: BMC Genomics. 2009 Mar 25;10:131. doi: 10.1186/1471-2164-10-131 (PMC2667191; doi:10.1186/1471-2164-10-131)

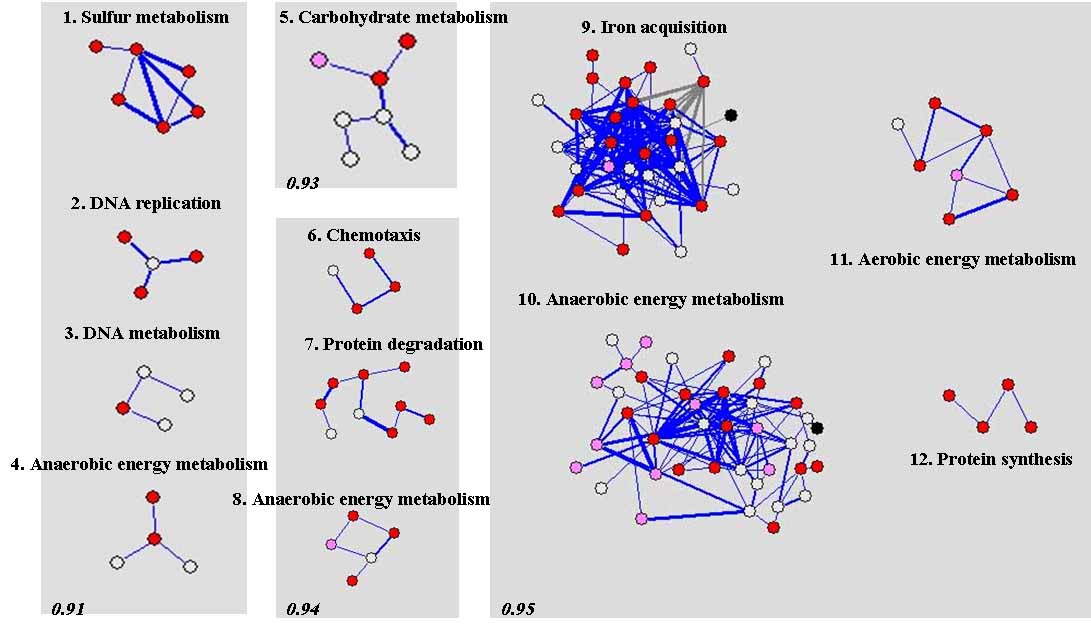

Supplement: Additional file 1 — Gene co-expression network inferred from the microarray data. Each node represents a gene and the width of line represents the correlation coefficient of two linked genes. Blue and gray lines indicate positive and negative correlation coefficients, respectively. Colors are assigned to nodes according to their functional categories: red represents the major functional category of each cluster, as indicated by text; lavender represents transcriptional regulator; white represents unknown genes and black nodes are genes whose functional links to other genes are not yet understood. The italic bold numbers are the cutoffs used to isolate clusters. [file 1471-2164-10-131-S1.jpeg]
